# Evolving Trends and Research Hotspots in Disaster Epidemiology From 1985 to 2020: A Bibliometric Analysis

**DOI:** 10.3389/fpubh.2021.720787

**Published:** 2021-08-30

**Authors:** Tao Liu, Xin Liu, Yue Li, Shuyu Liu, Chunxia Cao

**Affiliations:** Institute of Disaster and Emergency Medicine, Tianjin University, Tianjin, China

**Keywords:** disaster epidemiology, bibliometric analysis, Scopus, web of science, hotspots, trends

## Abstract

**Background:** Disaster epidemiology has not attracted enough attention in the past few decades and still faces significant challenges. This study aimed to systematically analyze the evolving trends and research hotspots in disaster epidemiology and provide insights into disaster epidemiology.

**Methods:** We searched the Scopus and Web of Science Core Collection (WoSCC) databases between 1985 and 2020 to identify relevant literature on disaster epidemiology. The retrieval strategies were TITLE-ABS-KEY (disaster epidemiology) and TS = (disaster AND epidemiology). Bibliometrix, VOSviewer 1.6.6 and SigmaPlot 12.5 were used to analyze the key bibliometric indicators, including trends and annual publications, the contributions of countries, institutions, journals and authors, and research hotspots.

**Results:** A total of 1,975 publications were included. There was an increasing trend in publications over the past 35 years. The USA was the most productive country. The most frequent institutions and journals were Fukushima Medical University and Prehospital and Disaster Medicine. Galea S made significant contributions to this field. “Epidemiology” was the highest-frequency keyword. COVID-19 was highly cited after 2019. Three research hotspots were identified: (i) the short- and long-term adverse health effects of disasters on the population; (ii) COVID-19 pandemic and emergency preparedness; and (iii) disaster management.

**Conclusions:** In recent decades, the USA was a global leader in disaster epidemiology. Disaster management, the short- and long-term health effects of disasters, and the COVID-19 pandemic reflected the research focuses. Our results suggest that these directions will remain research hotspots in the future. International collaboration is also expected to widen and deepen in the field of disaster epidemiology.

## Introduction

Disasters are one of the major threats facing our society's health today. A disaster is defined as “a serious disruption of the functioning of a community or a society causing widespread human, material, economic or environmental losses that exceed the ability of the affected community or society to cope using its own resources, thus necessitating a request to national or international level for external assistance” (1). In recent decades, the number of disasters has increased and led to, many deaths, injuries, diseases and disabilities (2, 3). Research on the health impacts of disasters has led to expanding use of epidemiological methods.

Epidemiology is the study of the distribution and determinants of disease and other health-related outcomes within populations (4). The application of epidemiology was applied in a disaster setting can produce estimates of the size of affected populations, quantify disaster-related morbidity, mortality and health outcomes (particularly the long-term effects), and provide evidence to demonstrate a causal relationship between exposures and health outcomes (5, 6). Disaster epidemiology is defined as the epidemiologic investigation of disaster forecasting and warning, emergency responses according to the different phases of disasters, and the short- and long-term adverse health effects of disasters on the population (7). Although disaster epidemiology is an evolving field, it still faces significant challenges (8).

Bibliometrics is a useful quantitative analysis approach to evaluate the quality and quantity of published papers (9) and can be used to explore the research trends, distribution of authorship, impact of publications and journals, and national and international contributions in a particular field (10–14). Bibliometric analysis has been applied to many fields, such as medicine, environmental health, computer science, and economics. Huang et al. (15) analyzed the state of research about the association of NO_2_, PM2.5 and noise exposure with cardiometabolic disorders. They identified three themes about research trends: the study of simultaneous exposures to multiple pollutants; the association between traffic-related pollutants and diabetes and metabolic symptoms; and the transition to the use of H-testing study designs to explore associations between noise and cardiometabolic outcomes. Guo et al. (16) provided a dynamic and longitudinal bibliometric analysis of healthcare-related artificial intelligence publications and reported that the major health problems studied in artificial intelligence research are cancer, depression, Alzheimer's disease, heart failure, and diabetes. Hao et al. (17) analyzed the development of disaster medicine to identify the main obstacles to improving disaster medicine research and application.

Disaster epidemiology scholars have published a substantial amount of research. However, the bibliometric profile of the disaster epidemiology literature is still unknown. Therefore, in this study, a bibliometric analysis was conducted to provide an overview of disaster epidemiology. This work identified current hotspots and estimated the contribution of leading countries, institutions, publishers, and researchers. The goal of the current study was to provide new perspectives for the further development of disaster epidemiology.

## Materials and Methods

### Data Sources and Search Strategy

We comprehensively searched the Scopus database and Web of Science Core Collection (WoSCC) database from 1985 to 2020 (5, 18, 19). Scopus is frequently used for bibliometric studies because, it is the largest abstract and citation database of peer-reviewed scientific literature (20, 21). We used the search strategy in the Scopus database as follows: [TITLE-ABS-KEY (disaster epidemiology) AND PUBYEAR > 1984 AND PUBYEAR <2021 AND (LIMIT-TO (DOCTYPE, “ar”) OR LIMIT-TO (DOCTYPE, “re”))]. Then, 1,686 publications related to disaster epidemiology were identified. WoSCC is an influential and multidisciplinary index database of academic literature abstracts worldwide that contains 10 sublibraries and is updated daily. We retrieved all documents from the WoSCC database using the following search strategy: [(TS = (disaster AND epidemiology) AND PY = 1985–2020)]. Regarding the document types, the search was restricted to only “article” and “review.” We retrieved 688 publications.

To avoid the bias caused by frequent database updates, we retrieved all literature and downloaded the data on the same day (March 14, 2021). Two investigators independently performed the search and had an agreement of 100% (*kappa* = 1 > 0.75), showing significant consistency (22). The expression was as follows:

$$\begin{array}{l} kappa\;=\;\left(P_{0}-P_{e}\right)\;/\;\left(n-P_{e}\right) \end{array}$$

In the present study, the inclusion and exclusion criteria were as follows: (i) all documents published between 1985 and 2020, including those published online, were included; (ii) only articles and reviews were included; (iii) all of the other document types, including editorial material, book reviews, retracted publications, proceedings papers, meeting abstracts, early access, corrections, news items, letters, book chapters and reprints, were excluded; and (iv) duplicate publications were excluded. Finally, a total of 2,374 publications were extracted from the two databases. The data, including titles, author information, abstracts, keywords, journals, and references, were downloaded in bib format.

### Data Analysis and Data Visualization

**Figure 1:**
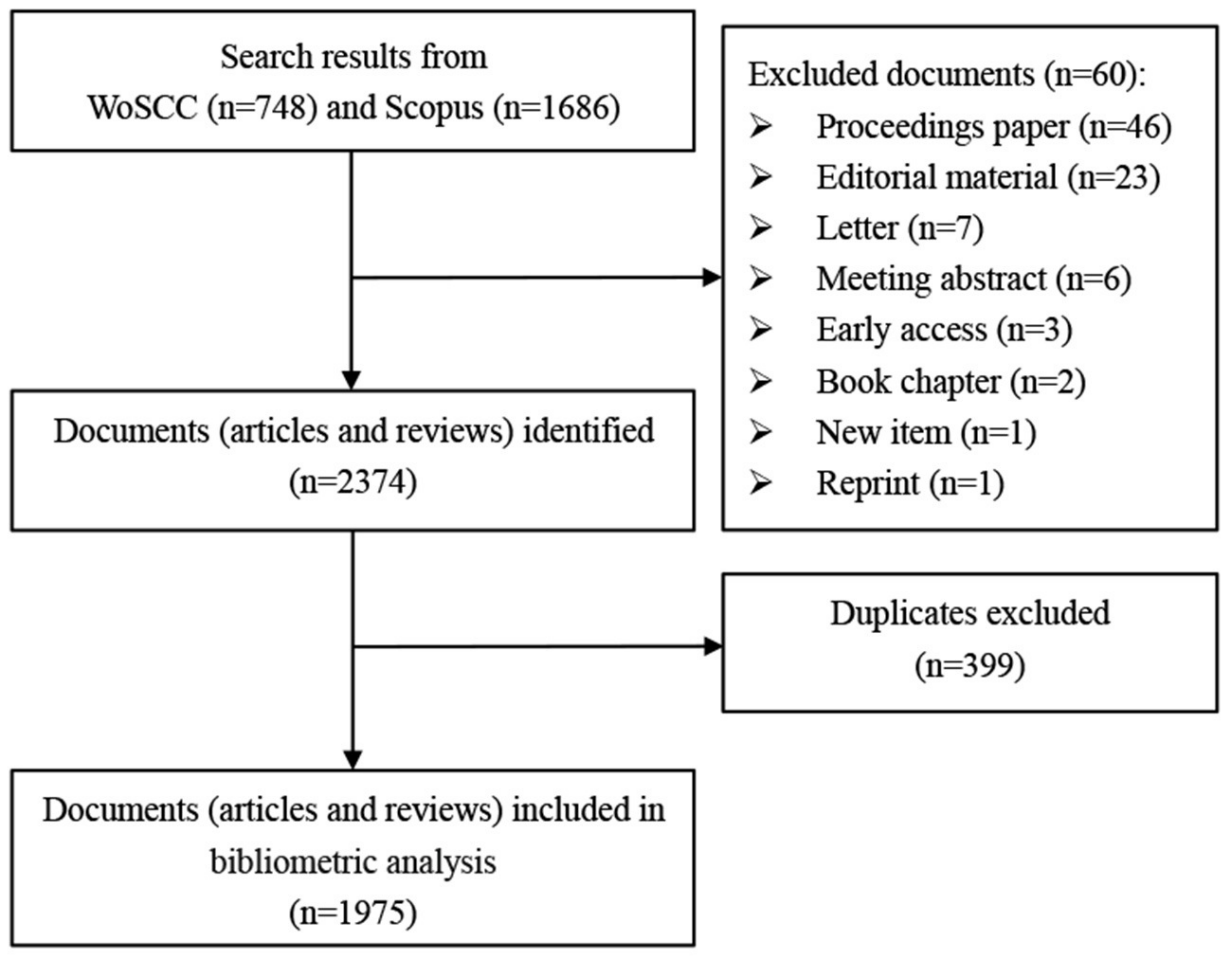
Flow chart detailing the paper collection and screening process. WoSCC, Web of Science Core Collection.

In the present study, the data analysis is divided into three substages. The first is to merge the databases. We merged the two databases and removed duplicate bibliographies (23–25). A total of 399 duplicates were deleted; therefore, the merged database included 1,975 publications (Figure 1). To control this process, we converted both WoSCC.bib and Scopus.bib to “bibtex” files. All analyses were performed using R (Version 4.0.4) and RStudio (Version 1.4.1106).

The second substage is descriptive analysis. The key bibliometric indicators were analyzed using Bibliometrix, an R package (23, 24), and they included the following: the annual trend of publications, most relevant keywords, most productive countries, journals, and authors, among others.

The final substage is data visualization. Sigmaplot (Version 12.5) was used to plot the time trend of publications. Map construction and visualization were conducted using the VOSviewer (Version 1.6.6) package program. In the network visualization, different colors represent different clusters, such as countries, authors and institutions; connecting lines represent parameters such as collaboration and cocitation. The size of the circle represents the magnitude of the correlation. The thickness of the connecting lines represents the collaboration strength (26–28).

## Results

### Trends and Annual Publications

Based on the merged databases, 1,975 publications (1,666 articles and 309 reviews) on disaster epidemiology from 1985 to 2020 were included in the final analysis, and 91.95% were in English (Supplementary Tables 1, 2).

**Figure 2:**
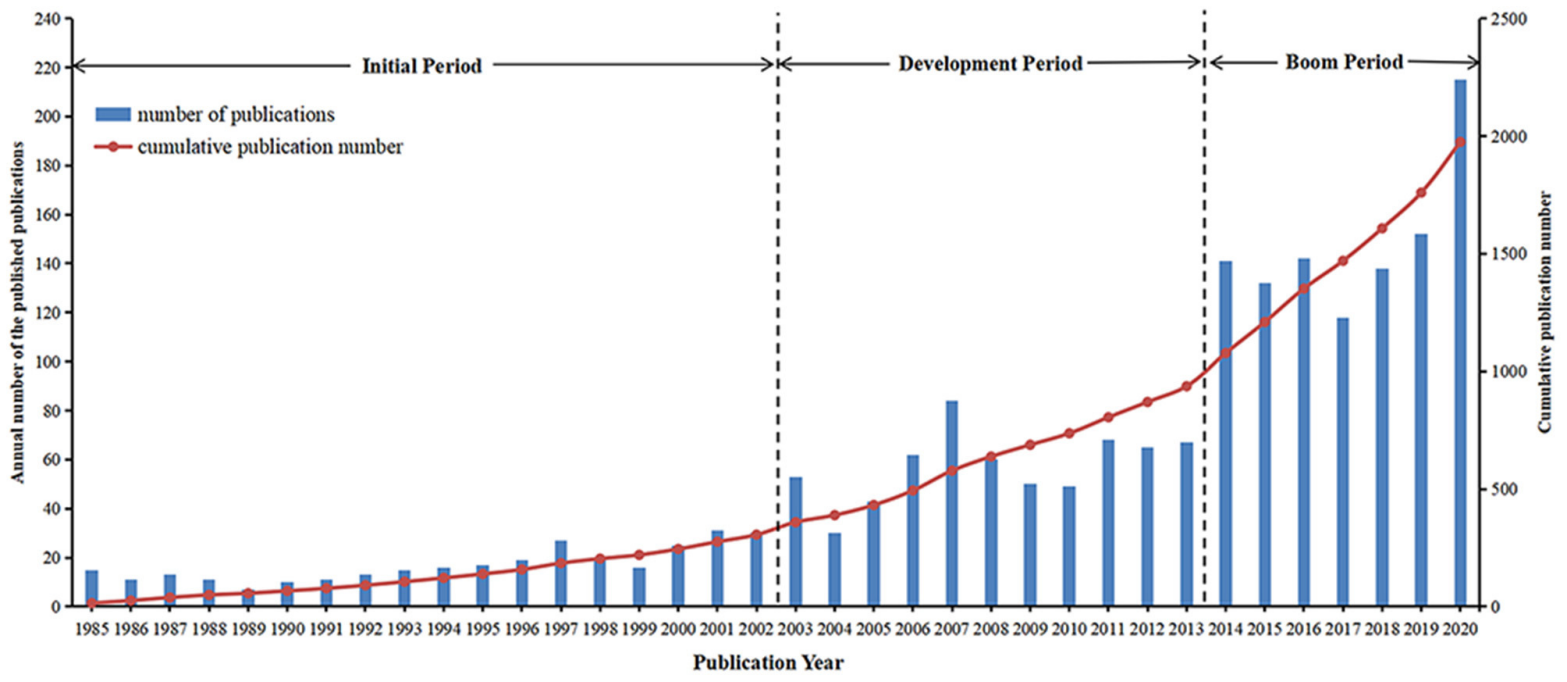
Annual number of the published publications and cumulative publication number on disaster epidemiology research from 1985 to 2020. “Annual number of published publications” is referred to the left axis and “cumulative publication number” to the right axis.

A vast increase in publication number was observed over this period, with annual publications growing from 15 in 1985 to 215 in 2020 (Figure 2). According to the publication number, this period was preliminarily divided into three stages: Stage 1, from 1985 to 2002, was considered as the initial period, when almost <30 papers were published annually and the average papers per year were 17.00; Stage 2, from 2003 to 2013, was known as the development period, with an average annual paper of 57.36, and the maximum number of annual publications was obtained in 2007 at 84; and. Stage 3, from 2014 to 2020, was the “boom period,” when the annual number of papers increased rapidly, with 1,038 documents being published (~53.00% of the total publications) and an annual average number of 148.29 (Figure 2). Among them, 38.60% (83/215) of the papers were related to disaster epidemiology and COVID-19 in 2020. Based on the Scopus and WoSCC databases, 1,975 papers were cited 41,302 times, and each paper was cited an average of 20.91 times.

### Contribution of Countries and Institutions

In this study, 86 countries and 3,554 institutions contributed to publications on disaster epidemiology between 1985 and 2020. Table 1 presents the top 10 most productive countries by the corresponding authors' origin. The USA published the greatest number of documents (654, 40.67%). Japan was the next leading country (126, 7.84%), followed by China (101, 6.28%). When countries were ranked based on multiple country publications (MCPs) (29), only the USA had a high percentage of papers. Publications from the USA had the highest share of citations (20,120, 55.32%). Publications from the Netherlands had the highest average citation per publication (30.87), followed by the USA (30.77).

**Table 1:** The top 10 countries contributing to publications on disaster epidemiology research.

**Country**	**Ranking based on total output**	**Output^**a**^, *n* (%)**	**SCP^**b**^**	**MCP^**c**^**	**Ranking based on citations**	**Citations^**d**^, *n* (%)**	**APC**
USA	1	654 (40.67)	620	34	1	20,120 (55.32)	30.77
Japan	2	126 (7.84)	123	3	3	1,582 (4.35)	12.56
China	3	101 (6.28)	96	5	8	1,059 (2.91)	10.49
United Kingdom	4	60 (3.73)	56	4	2	1,636 (4.50)	27.27
Australia	5	52 (3.23)	49	3	4	1,525 (4.19)	29.33
Italy	6	47 (2.92)	46	1	6	1,196 (3.29)	25.45
Canada	7	46 (2.86)	43	3	5	1,247 (3.43)	27.11
France	8	41 (2.55)	41	0	10	713 (1.96)	17.39
Iran	9	40 (2.49)	40	0	17	257 (0.71)	6.43
Netherlands	10	38 (2.36)	35	3	7	1,173 (3.23)	30.87

*APC, Average Publication Citations*.

a *N = 1,608*.

b *Articles in which all authors have the same country affiliation are called single country publications (SCP) and are considered to represent intra-country (within) collaboration*.

c *Articles with authors having different country affiliations are called multiple country publications (MCP) and considered to represent the international collaboration of that country*.

d *N = 36,370*.

**Figure 3:**
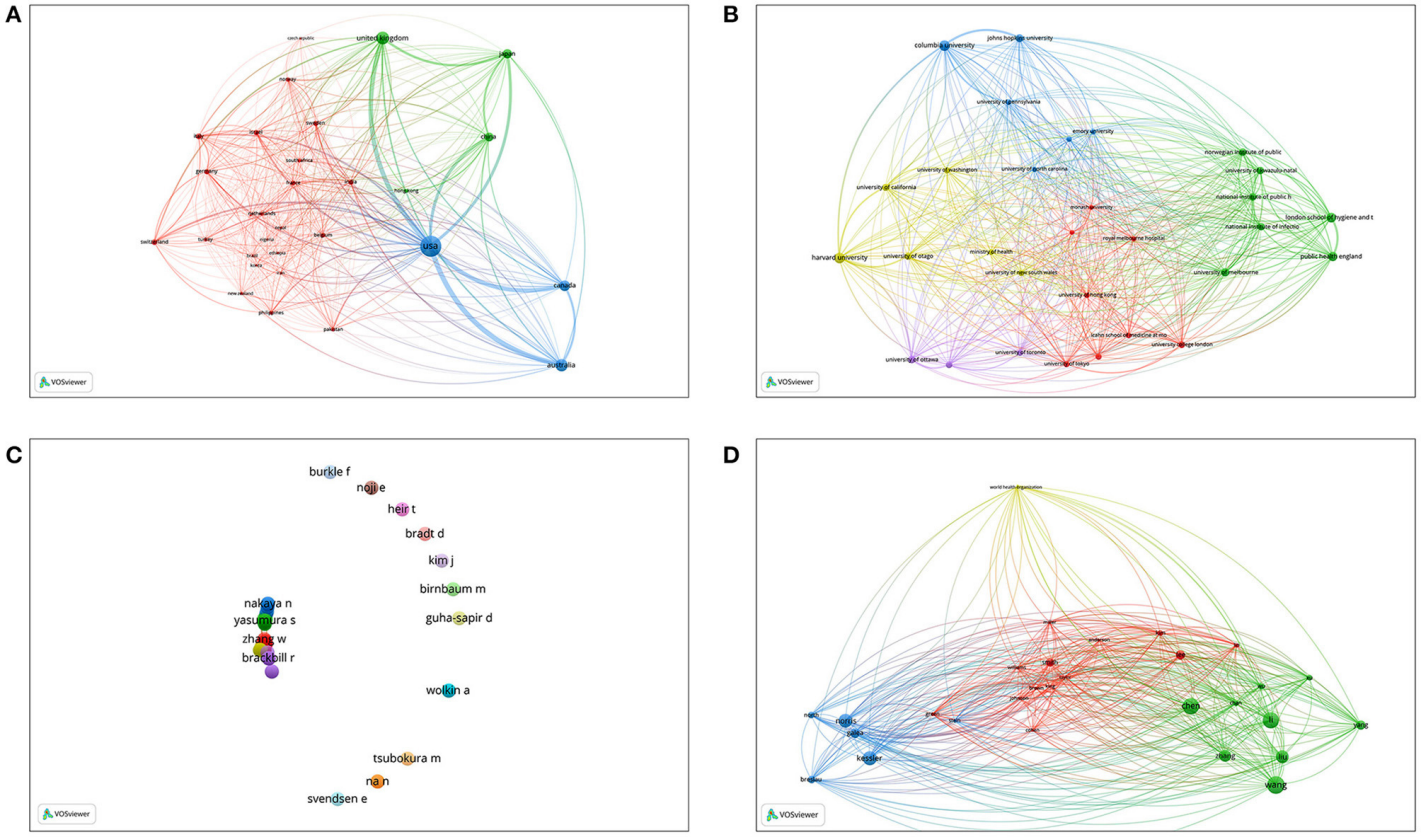
Network visualization map for **(A)** country collaboration, **(B)** institution collaboration, **(C)** author co-authorship, and **(D)** author co-citation.

The country coauthorship analysis indicated the degree of communication between the influential countries in this field (30). The map (Figure 3A) included 30 nodes. Researchers from the USA showed the highest collaboration performance, with a total link strength of 3,366, followed by the United Kingdom (total link strength = 1,242) and Australia (total link strength = 1,193).

Regarding the 10 most frequent institutions, *Fukushima Medical University* in Japan was the leading research institution, followed by *Tohoku University* in Japan and *California University* in the USA (Supplementary Table 3). Six are located in the USA, two are located in Japan, one is located in the Philippines, and one is located in Iran. Among all institutions included in the studies, 2,511 institutions published only one article, and 17 institutions published 20 or more articles (Supplementary Figure 1A). Approximately 60% of institutions have published 80% of publications in this field (Supplementary Figure 1B). The institution collaboration network is shown in Figure 3B. The most collaborative organizations included the following: *Columbia University* (total link strength= 72), *Harvard University* (total link strength= 70), and *London School of Hygiene and Tropical Medicine* (total link strength= 65), among others.

### Contribution of Journals

From 1985 to 2020, 903 journals contributed to the disaster epidemiology field. We comprehensively analyzed the characteristics of the top 10 most productive journals, including journal titles, article counts, the percentage of articles, CiteScore (2019), impact factor (IF) (2019), quartile in category (2019), and Hirsch index (h-index). As demonstrated in Table 2, these journals published 427 papers, accounting for 21.62% of the total publications. *Prehospital and Disaster Medicine* published the most papers, followed by *Disaster Medicine and Public Health Preparedness* and *International Journal of Environmental Research and Public Health*. The *American Journal of Public Health* had the highest IFs of any journals in 2019. The highest CiteScore also belonged to the *American Journal of Public Health*. The highest h-index was *PLoS One*. Among these, only the *American Journal of Public Health* was classified as Q1 according to the JCR 2019 standards.

**Table 2:** The top 10 most active journals that published articles on disaster epidemiology research.

**Rank**	**Journal**	**Article counts**	**Percent (%)**	**CiteScore**	**IF (2019)**	**Q (2019)**	**H-index**
1	Prehospital and Disaster Medicine	131	6.63	1.7	1.315	Q3	43
2	Disaster Medicine and Public Health Preparedness	105	5.32	1.8	0.977	Q4	33
3	International Journal of Environmental Research and Public Health	38	1.92	3.0	2.849	Q2	78
4	American Journal of Infection Control	32	1.62	4.1	2.294	Q2	97
5	Annals of Burns and Fire Disasters^a^	23	1.16	–	–	–	–
6	PLoS One	23	1.16	5.2	2.740	Q2	268
7	Disasters	20	1.01	3.2	1.937	Q2, Q3	61
8	BMJ Open	19	0.96	3.5	2.496	Q2	69
9	American Journal of Public Health	18	0.91	6.6	6.464	Q1	236
10	BMC Public Health	18	0.91	3.9	2.521	Q2	117

*IF, Impact Factor; Q, Quartile, with Q1 being the best in quality and Q4 being the least in quality; H-index, Hirsch index*.

a *no CiteScore, Impact Factor, Quartile in category and Hirsch index*.

The top 10 most highly cited publications contributed to 13.74% (5,674/41,302) of the total citations (Supplementary Table 4). The papers published in the *Journal of Consulting and Clinical Psychology, New England Journal of Medicine*, and *Epidemiologic Reviews* were the most cited (1,443 vs. 921 vs. 737 citations, respectively). Of the top 10 highly cited papers, two were published in the *New England Journal of Medicine* (IF = 74.699), and one was published in the Lancet (IF = 60.392) (31–33).

### Analysis of Research Hotspots

**Figure 4:**
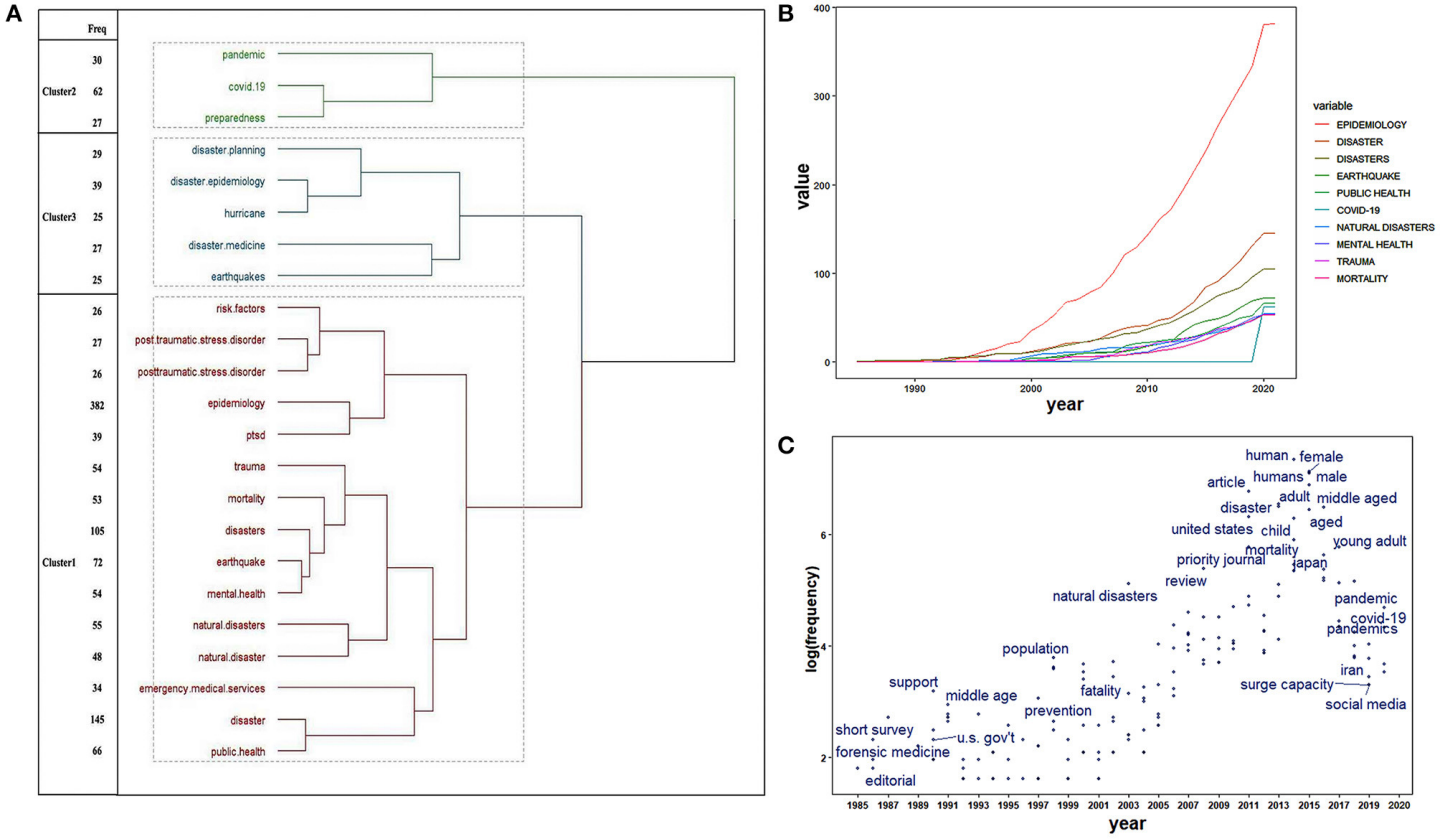
Research hotspot tendencies in the field of disaster epidemiology. **(A)** Topic dendrogram based on author keywords. **(B)** The occurrences of top author keywords annually. **(C)** The trend topics based on the keywords-plus.

A topic dendrogram (Figure 4A), trends of top keywords (Figure 4B), and topic trend plot (Figure 4C) were generated. With an appearance of more than 25 times, the 23 most frequent keywords were extracted from the included publications. Of these, the top 10 keywords are listed in Table 3. “Epidemiology” and “disaster(s)” were the most frequent keywords, followed by “earthquake” and “public health.” Figure 4B documents the J-shaped curves for “epidemiology” and “disaster(s),” with sustained growth from 1985 to 2020. The most recent was COVID-19, with a frequency of 62, which was highly cited after 2019 (Figure 4B; Table 3).

**Table 3:** Top 10-most relevant keywords.

**Rank**	**Author keywords**	**Frequency**
1	Epidemiology	382
2	Disaster(s)	250
3	Earthquake	72
4	Public Health	66
5	COVID-19	62
6	Natural Disasters	55
7	Mental Health	54
8	Trauma	54
9	Mortality	53
10	Natural Disaster	48

To further explore the changes in hotspots, the above 23 high-frequency keywords were sorted into three clusters using the R package. The representative articles involved in each cluster were mined to further summarize hotspots in the field of disaster epidemiology. Finally, 3 hotspots were identified (Figure 4A):

Cluster 1: Short- and long-term adverse health effects of disasters on the population;

Cluster 2: COVID-19 pandemic and emergency preparedness;

Cluster 3: Disaster management.

Hotspots have shifted from natural disasters to human health effects (e.g., mortality, posttraumatic stress disorder, etc.) exposure to natural disasters and man-made disasters. The most recent research hotspots were COVID-19 and the pandemic (Figure 4C). After the initial emergency responses are deployed to control the COVID-19 pandemic, we should start thinking about long-term strategies and concepts for pandemic and disaster governance, such as resilience.

### Contribution of Authors

In the present study, a total of 8,139 authors were counted. In terms of the most productive authors, *Galea S* ranked first with 24 publications, followed by *Rebmann T* with 20 publications and *Li J* with 18 publications (Supplementary Table 5). *Galea S* also had the highest local citations of 43. However, *Na N* ranked first as the first author, and *Galea S* ranked fourth among others.

Figure 3 also shows the network visualization map for author coauthorship and author cocitation on disaster epidemiology. *Yasumura S* had the largest total link strength of 279 (Figure 3C). Remarkably, the cooperation between the authors was relatively weak on the whole. *Wang* also made significant contributions to the field of disaster epidemiology, with the largest total link strength of 9,634 and links of 27 (Figure 3D).

## Discussion

During the present information explosion, bibliometric analysis can help scientific researchers visualize knowledge structures and recognize hotspots in a research field (34–36). Our study provides the most upto-date analysis of annual publication trends, the contribution of countries, institutions, journals and authors, and the research hotspot tendencies related to disaster epidemiology from 1985 to 2020.

### Trends and Annual Publication

A relatively slow increase in disaster epidemiology was observed from 1985 to 2020. However, since 2003, the pace of moving forward in this field has experienced the first shift. In 2014, the research ushered in a new era. The cause of these shifts was multifactorial. Disaster epidemiology may date to the 1970s. Epidemiological principles and methods began to be used in disaster response in the 1980s and had a general picture in the 1990s (5). Severe acute respiratory syndrome (SARS), defined as a disaster, broke out in late 2002 and early 2003, sickened more than 8,000 people, and spread to more than 30 countries within 6 months (37–39). The SARS pandemic has drawn the world's further attention to disaster epidemiology. Ten years later, Middle East respiratory syndrome (MERS) emerged in Saudi Arabia and spread to ~27 different countries with a fatality rate of 37% (40–42). The novel coronavirus (COVID-19) epidemic is a newly emerging infectious disease that was identified in China in late 2019, rapidly spreading to many countries and posing a major threat to public health (43–45). To explore the epidemic and impact of COVID-19, epidemiological methods were applied to model the COVID-19 pandemic (46, 47). This is why research on disaster epidemiology has increased rapidly in 2020. The epidemic and impact of COVID-19 also belong to the research scope of disaster epidemiology. This disaster highlighted the paramount importance of the practical application of epidemiological methods (48).

According to the growing trend of disaster epidemiology research, the publication volume will continue to grow linearly until the theory is mature. With the advent of COVID-19, we can estimate that research in this field will experience a large leap in the next several years (49).

### Publication Patterns

The field of disaster epidemiology has attracted people from all around the world, and developed countries are the main driving force, while developing countries have a limited effect. Multiple barriers, including funding, prioritization, research capacity, infrastructure, and language, contribute to these disproportionate results. Developing countries have limited resources, while developed countries allocate greater resources to this area and draw more interest from scientists. The USA contributed to most of the research and was the most active country and closely cooperated with many countries, playing an irreplaceable leading role in the field of disaster epidemiology. To shed light on the most active institutions, journals, and authors of a field are essential. Tracking the research trends of these institutions, journals, and authors will enable us to quickly grasp the research frontiers on disaster epidemiology (50). Collaborative regions, institutions, and authors were correlated geographically (51). Nevertheless, there is a lack of international cooperation with each other. The collaboration between authors is weaker, with no obvious major specialist groups. Under the general trend of cooperation, it is necessary for us to strengthen interdisciplinary and multidisciplinary cooperation. The investigation of top-cited papers can certainly help track high-impact journals, which identify research directions and complement the current body of knowledge. It is worth mentioning that the top-cited papers were focused on traumatic catastrophic events and their impact.

### Analysis of Research Hotspots

Based on the growing number of disaster epidemiology publications, the theoretical system, and methods of disaster epidemiology are gradually improving. Keywords reflect the concerns of the authors and their papers, which provides a general idea of research activity (52–54). The main domains of disaster epidemiology were epidemiology, disaster(s), earthquakes, public health and COVID-19. It is urgent to comprehensively assess the national health burden in disasters (e.g., earthquakes, floods, new infectious diseases) (55). Given the destructive and potential impacts of natural disasters, the threat of terrorism and the sheer unpredictable variants, disaster epidemiology can play an important role in controlling and mitigating the disaster's effects (55–58).

Disaster epidemiological methods can provide information about the health effects as well as resource allocation of social and community, manage the reports of social media, gauge medical needs, and assess impacts on health care systems in disaster settings (59). These methods have been applied to assess the scope and distribution of public health problems. Collecting epidemiological information in real time and practicing epidemiological methods throughout disasters contributes to disaster rescue and reduction of health burden (5).

Nearly all disasters carry a substantial public health risk and require both immediate and long-term assessment of their health effects on the population (55, 60, 61). However, significant challenges remain in disaster epidemiology as an evolving field (62). The COVID-19 pandemic has thrust epidemiologists and epidemiological models into the policy and media spotlight like, as never before, which highlights the importance of disaster epidemiology (56, 63). With the expansion of research activities, disaster epidemiology has also been further enriched. Bibliometric analysis may be a significant guide for tracking the growth in disaster epidemiology (64).

In the last 35 years, the times and lengths of citation bursts of each research topic on disaster epidemiology have varied. Certain keywords were extraordinarily consistent for a long period, while some keywords only briefly surged (16). Our study found that epidemiology and disaster(s) consistently acquired a high focus from 1985 to 2020. The COVID-19 pandemic, which broke out at the end of 2019, led to the dawn of a new era for the disaster epidemiology field (65). The frequency of COVID-19 in the literature was sharply increased.

Disaster epidemiology research has experienced several shifts in recent decades and continues to shift. We found that research hotspots have shifted from natural disasters to human health effects (e.g., mortality, posttraumatic stress disorder, etc.) exposure to natural disasters and man-made disasters. The most recent research hotspots were COVID-19 and the pandemic. The sustainability of research clusters related to disaster epidemiology is affected by the development of emergencies and public health events. Research related to disasters (e.g., earthquakes, floods, etc.) management and the short- and long-term adverse health effects of disasters on the population sustained a hotspot over the past decades, whereas some clusters were relatively short-lived, such as SARS. In particular, the COVID-19 pandemic and emergency preparedness have remained research hotspots since 2019. These research domains exert strong impacts on the field, and their influence will likely continue in the next few years (49). This information will provide directions for advancing the development of disaster epidemiology.

### Limitations

This study presented the bibliometric data from 1,975 publications on disaster epidemiology extracted from the merged database (WoSCC database and Scopus database) between 1985 and 2020. Even though the data analysis of this study was relatively objective and comprehensive, it also has limitations. First, bibliometric data change with time, and different conclusions may be drawn with time. Therefore, this study should be updated in the future. Second, there exists a discrepancy between the results of bibliometric analysis and the real research situation, which is due to the database remaining open as it continuously updates studies. In this case, the bibliometric analysis may not reflect the real situation.

## Conclusions

This study aimed to provide a basic overview of research publications on disaster epidemiology published between 1985 and 2020. The number of publications presented a trend of continuous growth, and developed countries are the main force of this field. The hotspots of research on disaster epidemiology are closely related to the development of emergencies and public health events. Research related to disaster management and the short- and long-term adverse health effects of disasters on the population will be hotspots. The COVID-19 pandemic and emergency preparedness will also gain more attention. These research domains will likely continue in the next few years and exert strong impacts on advancing the development of disaster epidemiology. These results provide new perspectives for the study of disaster epidemiology and may have a beneficial effect on further study regarding the development of disaster epidemiology and possible practice implications.

## Data Availability Statement

The original contributions presented in the study are included in the article/Supplementary Material, further inquiries can be directed to the corresponding author/s.

## Author Contributions

CC and TL conceptualized and designed the study and drafted the article. XL and TL independently performed the search databases. TL, YL, and SL contributed to the analysis and interpretation of data. CC, TL, and YL reviewed and revised the article critically for important intellectual content. CC takes responsibility for the paper as a whole. All authors approved the final article and agreed to be accountable for all aspects of the work.

## Conflict of Interest

The authors declare that the research was conducted in the absence of any commercial or financial relationships that could be construed as a potential conflict of interest.

## Publisher's Note

All claims expressed in this article are solely those of the authors and do not necessarily represent those of their affiliated organizations, or those of the publisher, the editors and the reviewers. Any product that may be evaluated in this article, or claim that may be made by its manufacturer, is not guaranteed or endorsed by the publisher.
