# Supplementary material for: Evolving Trends and Research Hotspots in Disaster Epidemiology From 1985 to 2020: A Bibliometric Analysis
Source: Front Public Health. 2021 Aug 30;9:720787. doi: 10.3389/fpubh.2021.720787 (PMC8435596; doi:10.3389/fpubh.2021.720787)
Supplement: Supplementary file 1 [file Data_Sheet_1.docx]

Supplementary Material

# Supplementary Figures and Tables

## Supplementary Tables

**Supplementary Table 1.** Main information regarding the collection.

| **Description** | | |
| --- | --- | --- |
| Main information about data | Period | 1985-2020 |
|  | Documents | 1975 |
|  | Average citations per documents | 20.91 |
|  | Average citations per year per documents | 1.90 |
|  | References | 62667 |
|  | Countries | 86 |
|  | Institutions | 3554 |
|  | Sources (Journals, Books, etc) | 903 |
| Document types | Article | 1666 |
|  | Review | 309 |
| Document contents | Keywords Plus | 7900 |
|  | Author’s Keywords | 3554 |
| Authors | Authors | 8139 |
|  | Author Appearances | 10358 |
|  | Authors of single-authored documents | 248 |
|  | Authors of multi-authored documents | 7891 |
| Authors collaboration | Single-authored documents | 285 |
|  | Documents per Author | 0.24 |
|  | Authors per Document | 4.12 |
|  | Co-Authors per Documents | 5.24 |
|  | Collaboration Index | 4.67 |

**Supplementary Table 2.** The information of publication languages (total=1975).

| **Rank** | **Language** | | **Documents** | **Percent (%)** |
| --- | --- | --- | --- | --- |
| 1 | | English | 1816 | 91.95 |
| 2 | | French | 36 | 1.82 |
| 3 | | Spanish | 25 | 1.27 |
| 4 | | German | 19 | 0.96 |
| 5 | | Chinese | 17 | 0.86 |
| 6 | | Russian | 15 | 0.76 |
| 7 | | English; Spanish | 7 | 0.35 |
| 8 | | English; Ukrainian | 6 | 0.30 |
| 9 | | Italian | 5 | 0.25 |
| 10 | | Japanese | 4 | 0.20 |
| 11 | | Dutch | 3 | 0.15 |
| 12 | | English; Portuguese | 3 | 0.15 |
| 13 | | Polish | 3 | 0.15 |
| 14 | | Czech | 2 | 0.10 |
| 15 | | English; French | 2 | 0.10 |
| 16 | | Korean | 2 | 0.10 |
| 17 | | Lithuanian | 2 | 0.10 |
| 18 | | Persian | 2 | 0.10 |
| 19 | | Portuguese | 2 | 0.10 |
| 20 | | Croatian | 1 | 0.05 |
| 21 | | Greek | 1 | 0.05 |
| 22 | | Hebrew | 1 | 0.05 |
| 23 | | Romanian; Moldavian; Moldovan | 1 | 0.05 |

**Supplementary** **Table 3.** Top 10-most frequent institutions (of all co-authors for each paper).

| **Rank** | **Institutions** | **Country** | **Frequency** |
| --- | --- | --- | --- |
| 1 | Fukushima Medical University | Japan | 77 |
| 2 | Tohoku University | Japan | 49 |
| 3 | California University | USA | 33 |
| 4 | Columbia University | USA | 30 |
| 5 | Johns Hopkins University | USA | 30 |
| 6 | Harvard University | USA | 29 |
| 7 | Saint Louis University | the Philippines | 28 |
| 8 | Centers for Disease Control and Prevention | USA | 26 |
| 9 | Icahn School of Medicine at Mount Sinai | USA | 26 |
| 10 | Tehran University of Medical Sciences | Iran | 25 |

**Supplementary** **Table 4.** Top 10-most cited papers.

| **Rank** | **Author, year** | **Document title** | **Journal** | **TC** | **TCY** | **TD** |
| --- | --- | --- | --- | --- | --- | --- |
| 1 | Resnick HS, 1993 | Prevalence of civilian trauma and posttraumatic stress disorder in a representative national sample of women | J Consult Clin Psychol | 1443 | 49.76 | Article |
| 2 | Helzer JE, 1987 | Post-traumatic stress disorder in the general population. | New Engl J Med | 921 | 26.31 | Article |
| 3 | Galea S, 2005 | The epidemiology of post-traumatic stress disorder after disasters | Epidemiol Rev | 737 | 43.35 | Review |
| 4 | Breslau N, 1997 | Sex differences in posttraumatic stress disorder | Arch Gen Psychiatry | 452 | 18.08 | Article |
| 5 | Mccord C, 1990 | Excess mortality in harlem | New Engl J Med | 420 | 13.12 | Article |
| 6 | Jonkman SN, 2005 | Global perspectives on loss of human life caused by floods | Nat Hazards | 386 | 22.71 | Article |
| 7 | Breslau N, 2000 | A second look at comorbidity in victims of trauma: the posttraumatic stress disorder-major depression connection | Biol Psychiatry | 367 | 16.68 | Article |
| 8 | Wang H, 2017 | Global, regional, and national under-5 mortality, adult mortality, age-specific mortality, and life expectancy, 1970-2016: a systematic analysis for the Global Burden of Disease Study 2016 | Lancet | 328 | 65.60 | Article |
| 9 | Peters DPC, 2004 | Cross-scale interactions, nonlinearities, and forecasting catastrophic events | Proc Natl Acad Sci U S A | 312 | 17.33 | Article |
| 10 | Barredo JI, 2007 | Major flood disasters in Europe: 1950-2005 | Nat Hazards | 308 | 20.53 | Article |

TC Total Citations; TCY Total Citations per Year; TD Type of Document

**Supplementary** **Table 5.** The top 10 most productive authors on disaster epidemiology research.

| Authors | Ranking based on article counts | Articles Counts | | Authors | Ranking based on local citations | | Local Citations^a^ | | First Author^b^ | Ranking based on articles fractionalized | | Articles Fractionalized^c^ |
| --- | --- | --- | --- | --- | --- | --- | --- | --- | --- | --- | --- | --- |
| Galea S | 1 | 24 | Galea S | | | 1 | | 43 | Na N | | 1 | 14.00 |
| Rebmann T | 2 | 20 | Vlahov D | | | 2 | | 37 | Noji E | | 2 | 8.00 |
| Li J | 3 | 18 | Nandi A | | | 3 | | 36 | Rebmann T | | 3 | 6.85 |
| Yasumura S | 4 | 16 | Peek-Asa C | | | 4 | | 21 | Galea S | | 4 | 5.78 |
| Suzuki Y | 5 | 15 | Ramirez M | | | 5 | | 16 | Bradt D | | 5 | 3.75 |
| Na N | 6 | 14 | Wang H | | | 6 | | 11 | De B J | | 6 | 3.00 |
| Brackbill R | 7 | 12 | Espinel Z | | | 7 | | 9 | Diaz J | | 7 | 3.00 |
| Wang J | 8 | 12 | Helzer J | | | 8 | | 9 | Rezaeian M | | 8 | 3.00 |
| Bromet E | 9 | 11 | Mcevoy L | | | 9 | | 9 | Yasumura S | | 9 | 2.84 |
| Cone J | 10 | 11 | Robins L | | | 10 | | 9 | Heir T | | 10 | 2.83 |

^a^measures how many times an author included in this field have been cited by the documents also included in the field

^b^is corresponding author of each manuscript

^c^means the authors’ frequency distribution (fractionalized)

## Supplementary Figures


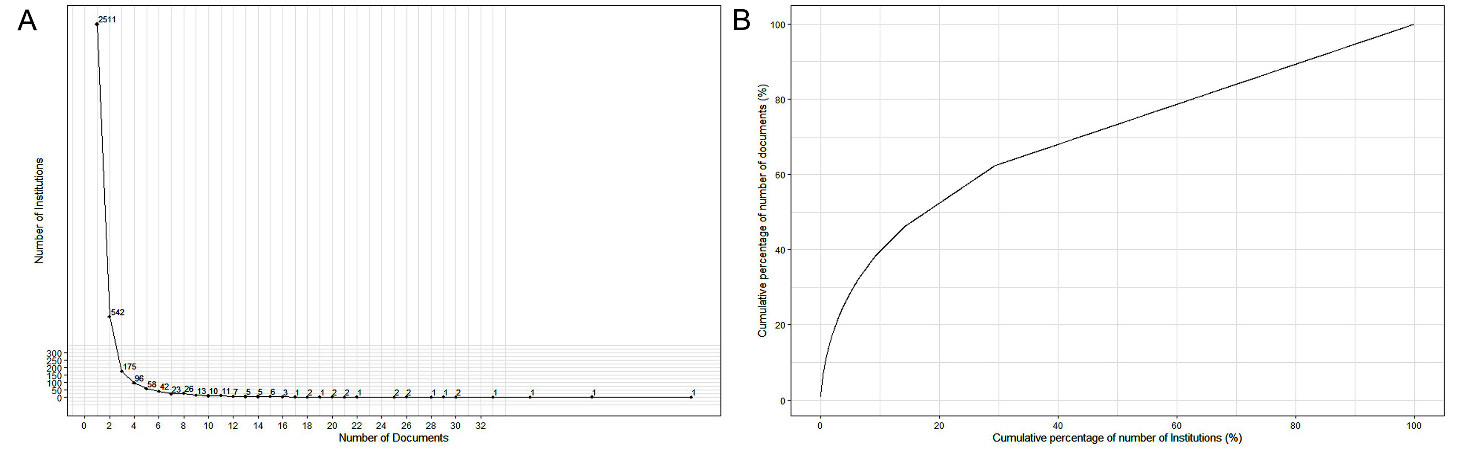


**Supplementary Figure 1.** (A) The relationship between the number of institutions and the number of documents. (B) The relationship between the cumulative percentage of institutions number and the cumulative percentage of documents number.
